# Genome-resolved metagenomics reveals potential biochemical capabilities of a microbial community in an industrial wastewater membrane bioreactor during performance deterioration and recovery

**DOI:** 10.3389/fmicb.2026.1908813

**Published:** 2026-10-09

**Authors:** Hana Barak, Asher Brenner, Ariel Kushmaro

**Affiliations:** 1 Avram and Stella Goldstein-Goren Department of Biotechnology Engineering, Ben-Gurion University of the Negev, Beer-Sheva, Israel; 2 Department of Civil and Environmental Engineering, Ben-Gurion University of the Negev, Beer-Sheva, Israel; 3 The Ilse Katz Center for Nanoscale Science and Technology, Ben-Gurion University of the Negev, Beer-Sheva, Israel; 4 School of Sustainability and Climate Change, Ben-Gurion University of the Negev, Beer-Sheva, Israel

**Keywords:** bioremediation, industrial wastewater, MBR, metagenome-assembled genomes, metagenomics

## Abstract

This study investigates the microbial community composition and biochemical capabilities within a membrane bioreactor (MBR) system treating industrial wastewater during two operational states: a period of marked performance deterioration and a subsequent early recovery phase. The MBR system experienced a prolonged decline in treatment performance, with Total Organic Carbon (TOC) removal efficiency decreasing from approximately 90% to approximately 70% and effluent Biochemical Oxygen Demand (BOD) increasing to more than 70 mg/L. Metagenomic sequencing and subsequent analysis unveiled distinct shifts in microbial community composition and functional potential during these operational states. A total of 51 Metagenome-Assembled Genomes (MAGs) were reconstructed and characterized, revealing microbial populations associated with *Pseudomonadota* and *Bacteroidota*. These populations exhibited putative metabolic capabilities associated with nitrogen cycling, sulfur metabolism, and degradation of recalcitrant aromatic compounds. Key taxa enriched during the early recovery condition contained genes associated with nitrogen fixation and sulfur oxidation. These findings provide genome-resolved insights into microbial community composition and predicted functional potential during contrasting MBR operational states and may inform future investigations of industrial wastewater treatment systems.

## 1 Background

Industrial wastewater is considered to be one of the most crucial sources of pollution to the environment due to the presence of toxic compounds (e.g., halogenated organics, phenolic compounds, aromatic amines, sulfides, cyanides, and more), high levels of organic matter, high salinity, and extreme physicochemical properties (e.g., pH, ORP; Mutamim et al., 2012). Therefore, industrial wastewater must be treated cautiously before it is discharged into receiving water bodies or reused.

Membrane bioreactors (MBRs) have emerged as efficient and sustainable solutions for industrial wastewater treatment due to their ability to combine biological treatment through activated sludge with membrane filtration, allowing for the continuous separation of biomass from the treated water (Rahman et al., 2023). In this process, microorganisms break down organic matter in the bioreactor, while the membrane separates the treated water from the biomass, preventing the loss of active microorganisms. This integrated approach results in improved pollutant removal and more stable effluent. Over the last two decades, MBR technologies have attracted considerable attention because of their ability to produce high-quality effluent and are now considered well-established technologies for wastewater treatment (Al-Asheh et al., 2021; Marrot et al., 2004). MBRs also offer advantages such as higher treatment efficiency and reduced footprint, in comparison to conventional treatment methods (Neoh et al., 2016). However, despite these advantages, MBRs are not immune to operational challenges that can compromise performance. Factors such as fluctuations in influent characteristics, membrane fouling, imbalances in microbial populations, and exposure to toxic compounds can lead to temporary performance crashes. For instance, variations in organic and inorganic loading, salinity, or oxygen levels can significantly impact the microbial community composition, which is integral to MBR functionality. Understanding these operational challenges and their effects on MBR performance is critical to ensuring system resilience and stability.

The arrangement and composition of microbial communities within bioreactors exhibit variability across different MBR plants and even over time within a single MBR unit. This variability primarily stems from the significant role that microorganisms play in these environmental engineering systems. The diverse microbial communities in bioreactors are influenced by the wastewater fed into the system, operating conditions, and reactor design (Al-Asheh et al., 2021; Nguyen et al., 2020).

MBR systems are susceptible to operational challenges and occasional failures that can significantly impact their performance and efficiency. Understanding the microbial community dynamics in MBRs both during normal operation and failure events is therefore crucial for optimizing system stability and recovery (Barak et al., 2020). Various techniques have been used to study microbial ecology in biological wastewater treatment. Traditional cultivation-based methods are limited in promoting a full understanding of the complex microbial communities in these systems. With only 1% of global wastewater microbial species being cultivable, there is a growing need for more direct and comprehensive approaches to gain insights into microbial communities in wastewater treatment (Chen et al., 2022).

Metagenomics is one useful method to learn about microbial communities in different environments. This approach directly sequences DNA fragments extracted from environmental samples, thereby avoiding biases associated with PCR amplification of specific marker genes and providing a broader representation of the microbial community. Indeed, metagenomic sequencing has emerged as a powerful tool for unraveling the microbial diversity, metabolic pathways, and potential interactions within the community in various environments, including biological wastewater treatment systems (Mardanov et al., 2025). Recent metagenomic work on activated sludge from a common effluent treatment plant receiving heterogeneous industrial wastewater demonstrated how shotgun sequencing can simultaneously resolve community structure and functional potential, including xenobiotic degradation and nutrient-cycling pathways (Vasudeva et al., 2022). Therefore, using a combination of metagenomic sequencing and advanced binning techniques allows for the construction of Metagenome-Assembled Genomes (MAGs). MAGs enable a more refined view of individual microbial populations within a community, allowing researchers to delve deeper into their genetic potentials and possible roles in ecosystems processes (Yang et al., 2021).

In the realm of industrial wastewater treatment, MBR success depends on the intricate biochemical capabilities orchestrated by the microbial communities within these systems. These microbial consortia play a vital role in driving essential biogeochemical cycles, such as the carbon, sulfur, and nitrogen cycles (Grubba et al., 2022). Additionally, these communities exhibit remarkable proficiency in the degradation of recalcitrant aromatic compounds (Mozo et al., 2011), showcasing their potential to address complex industrial wastewater challenges. Understanding the genetic foundations of these capabilities offers a window into the molecular mechanisms underpinning these vital processes, providing valuable insights for optimizing MBR performance and advancing our quest for effective and sustainable industrial wastewater treatment solutions.

In this study, we build upon our previous investigation focused on the temporal distribution of microbial community in an industrial wastewater treatment system following crash and during recovery periods (Barak et al., 2020). We delve into the analysis of the sequencing data derived from a chemical facility situated in the Neot Hovav Industrial Park, formerly known as Ramat Hovav, located in the Negev Desert, Israel. Through the use of metagenomic sequencing combined with binning techniques, this study reconstructed Metagenome-Assembled Genomes (MAGs), enabling a comprehensive exploration of the microbial community’s predicted biochemical and metabolic capabilities. The present investigation compared genome-resolved taxonomic and predicted functional traits between two sampled operational states of the Membrane Bioreactor. These findings provide a genome-resolved description of microbial populations and predicted functional traits associated with the two sampled MBR operational states and may guide future longitudinal and activity-based investigations.

## 2 Methods

### 2.1 System description and chemical analyses

Biomass samples were collected from a full-scale membrane bioreactor (MBR) treating wastewater generated by a major chemical factory in the Neot Hovav Industrial Area. The MBR system consists of four aeration basins, each with a volume of 6,000 m^3^. Typically, three basins are operational, while the fourth is reserved for emergency operations. Wastewater is continuously fed to the reactor at an average flow rate of approximately 2,000 m^3^/day, following a 3–5 day equalization period. The effluent is discharged from the system through an ultrafiltration membrane system, which retains the biomass on a membrane with a pore size of approximately 4 μm. To characterize process performance, daily measurements of total organic carbon (TOC), chemical oxygen demand (COD) and biochemical oxygen demand (BOD) were conducted. The December 2016 and February 2017 sampling dates were retrospectively designated as the crash and early recovery conditions, respectively, based on the monthly reactor-performance trends.

### 2.2 Sample collection and total genomic DNA extraction

One-liter biomass samples were directly collected from the MBR aeration basins in December 2016, representing the deteriorated (crash) condition, from reactors A, B, and C, and in February 2017, representing the early recovery condition, from reactors A, B, C, and D. The aeration basins were arranged sequentially within the same treatment system; therefore, samples collected from different reactors at each sampling date represent reactor-level observations nested within a single operational state rather than fully independent biological replicates. Total bacterial DNA was extracted from the samples using the PowerSoil DNA Isolation Kit (MO BIO Laboratories, San Diego, USA) following the manufacturer’s protocol with slight modifications. In empty PowerSoil bead tubes, 500 μL of each sample was centrifuged for 1 min, and the supernatant was discarded. The original contents of the PowerSoil bead tubes were then returned to the tubes, and the pellets were dissolved by vortexing. After adding 60 μL of C1 solution (Kit Component), the samples were homogenized by horizontal vortexing for 10 min. All subsequent steps were performed according to the manufacturer’s protocol.

### 2.3 Metagenomics—sequencing and analysis

The total genomic DNA extracted from the seven samples was submitted to the DNA Services (DNAS) Facility at the Research Resources Center, University of Illinois at Chicago (UIC), for whole-genome shotgun sequencing (WGS). The genomic DNA was processed using the Nextera XT library preparation kit and sequenced on the Illumina NextSeq500 instrument. For each sample, four sets of forward and reverse FASTQ files were generated.

Data were first processed using the NeatSeq Flow workflow platform (Sklarz et al., 2017) for general community profiling. Raw-read quality was assessed using FastQC (V0.11.8; Andrews, 2010). Adapter and quality trimming were performed using Trim Galore (V0.6.2; Krueger, 2015), with 15 nucleotides removed from the 5′ ends of both forward and reverse reads, a minimum Phred quality score of 25, and a minimum retained read length of 36 bp. Taxonomic profiling was performed using Kaiju (V1.7.0; Menzel et al., 2016) and functional analysis was carried out through HUMAnN2 (V0.11.2; Franzosa et al., 2018) using the UniRef90 (Suzek et al., 2007). Principal component analyses for the taxonomic and metabolic profiles were carried out using the JMP software (V14.3.0; Jones and Sall, 2011). Unless otherwise specified, software was run using default parameters.

For Metagenome-Assembled Genomes (MAGs) construction and characterization, the metagenomic data underwent processing using the metaWRAP pipeline (V1.2.1; Uritskiy et al., 2018) with default parameters, unless otherwise specified. Raw reads were quality controlled using TrimGalore (Trim Galore V0.5.0; Krueger, 2015) and low-quality reads were removed. The quality-controlled sequences from all samples were pooled and co-assembled using MetaSPAdes (Nurk et al., 2017). The resulting assemblies, along with the quality-controlled sequences, were used for metagenomic binning employing three different algorithms: MaxBin (V2.2.6; Wu et al., 2014), metaBAT (V2.12.1; Kang et al., 2015) and CONCOCT (V1.0.0; Alneberg et al., 2014). The bin sets were refined and consolidated using the MetaWRAP Bin_refinement module to obtain a more robust set with a minimum completeness of 50% and a maximum contamination of 10%. The consolidated bin set was reassembled using the MetaWRAP Reassemble_bins module. Reads were mapped with BWA-MEM (V0.7.17) and recruited using strict (perfect matches) and permissive (<3 mismatches) criteria. Each set was reassembled with SPAdes (V3.13.0; --careful) using the original bin as untrusted contigs. Contigs <1,000 bp were removed, and CheckM (V1.0.12) was used to select the best version based on completeness and contamination.

Taxonomic classification of the consolidated bins was performed using GTDB-Tk version 2.7.2 (Chaumeil et al., 2020) with the GTDB release R232. A maximum-likelihood phylogenomic tree was constructed from the GTDB-Tk bac120 concatenated marker-protein alignment using IQ-TREE (V3.1.2; Minh et al., 2020), with ModelFinder for model selection, 1,000 ultrafast bootstrap replicates, and 1,000 SH-aLRT replicates to assess branch support. The resulting tree was visualized and annotated in R using the ggtree package (V4.2.0). The consolidated bins were subsequently annotated using Prokka (V1.13; Seemann, 2014) through metaWRAP’s Annotate_bins module. Additional analyses of metabolic and biogeochemical functional traits were conducted using the METABOLIC profiler software (Zhou et al., 2022) with METABOLIC-C.pl version 4.0.

The abundances of the MAGs across samples were estimated using the metaWRAP Quant_bins module with default parameters. This module uses Salmon to quantify read abundance for individual contigs and calculates length-weighted average abundance for each bin. Abundance values are reported as genome copies per million reads, providing normalization for differences in sequencing depth among samples. To identify the differential abundance of Metagenome-Assembled Genomes (MAGs) between the crash and recovery periods, the linear discriminant analysis (LDA) effect size (LEfSe) algorithm was employed (Segata et al., 2011). LEfSe was used to determine the direction of enrichment and calculate the LDA effect size for each MAG. To account for multiple testing, Kruskal–Wallis *p*-values were calculated for all tested MAGs and adjusted using the Benjamini–Hochberg false discovery rate procedure. MAGs with an LDA score >3 and an adjusted *q*-value <0.05 were considered significantly enriched. Raw *p*-values, adjusted q-values, LDA scores, direction of enrichment, and abundance values for all MAGs are provided in Supplementary Tables S3, S4. The combined visualization of bin abundance and LDA effect size was generated in R (Ihaka and Gentleman, 1996) using the ggplot2 package (Wickham and Sievert, 2009).

## 3 Results

### 3.1 Reactor performance and definition of sampled operational states

Monthly performance of the bioreactor was evaluated using Total Organic Carbon (TOC) removal efficiency and mean effluent Biochemical Oxygen Demand (BOD) and Chemical Oxygen Demand (COD) concentrations from May 2016 to November 2017 (Figure 1). The performance results revealed a significant change in both TOC removal efficiency and BOD and COD concentrations, indicating a notable shift in the bioreactor’s operational state from August 2016 to January 2017, representing a prolonged deterioration period, hereafter referred to as the crash condition. The TOC removal efficiency percentage gradually declined during the crash period, decreasing from approximately 90% at the outset to around 70% by the time of the bioreactor’s collapse. This decline in TOC removal efficiency was concomitantly associated with a marked increase in the monthly average BOD and COD of the bioreactor effluent, indicating a significant failure in the biodegradation process. The mean BOD and mean COD concentrations, which were initially within an acceptable range, demonstrated a substantial rise, exceeding 70 mg/L and 3,000 mg/L, respectively, during the period of impaired reactor performance. Beginning in January 2017, the performance indicators showed a reversal of this deterioration trend, with increased TOC removal efficiency and decreased effluent BOD and COD concentrations. Accordingly, the February 2017 sampling date was defined as an early recovery condition. However, the available data do not demonstrate that the system had fully returned to its stable pre-crash baseline.

**Figure 1:**
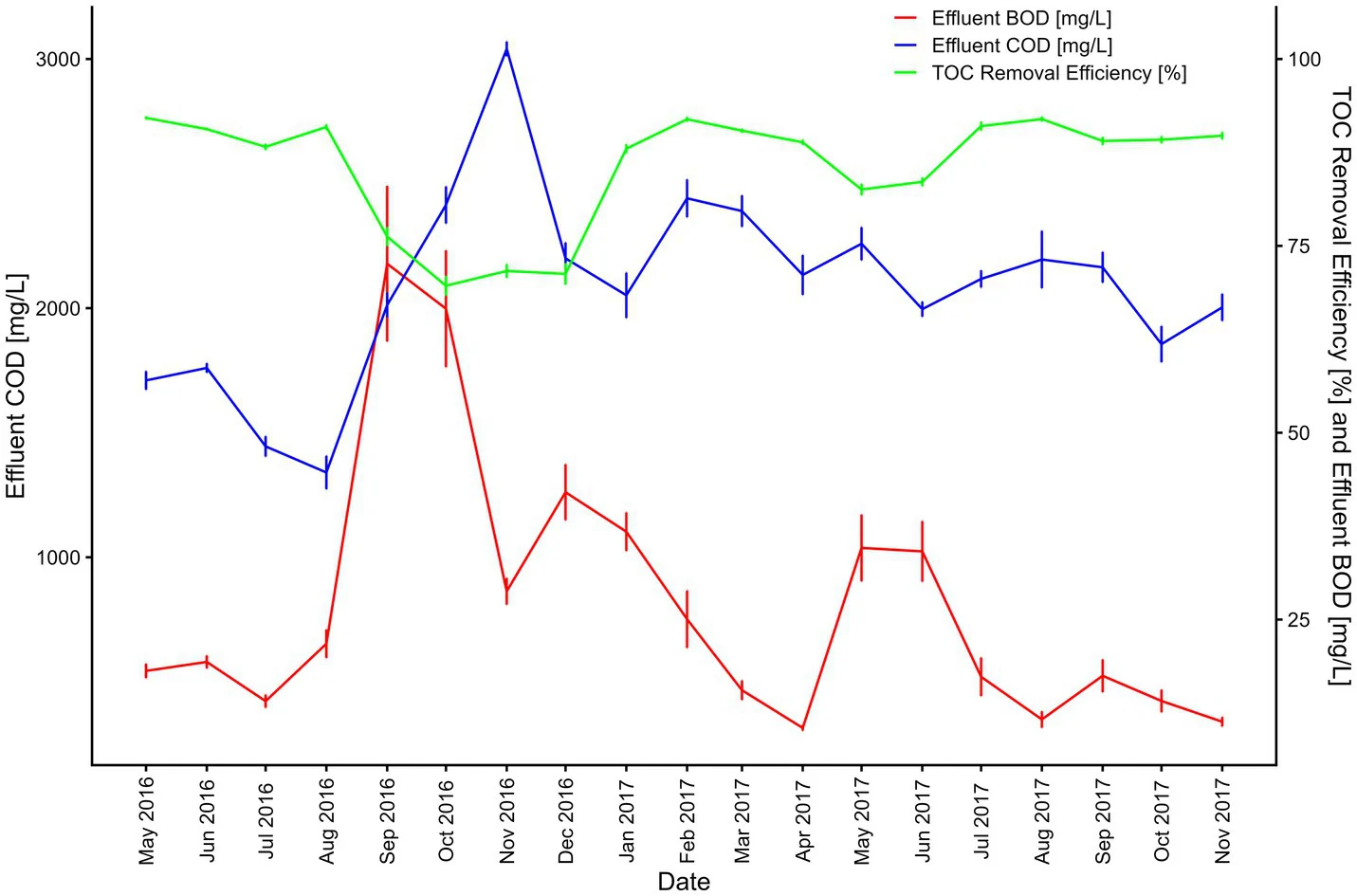
Average monthly values of TOC removal efficiency [%] (green), BOD (red), and COD (blue) in the bioreactor effluent [mg/L], with error bars (SE).

Mean concentrations of Total Organic Carbon (TOC), Chemical Oxygen Demand (COD), and Total Dissolved Solids (TDS) in the raw wastewater were initially measured in order to evaluate the bioreactor’s performance. These baseline measures were recorded at 2,707 mg/L, 9,585 mg/L, and 21,987 mg/L, respectively.

### 3.2 Sequencing output and community-level profiles

To gain deeper insights into the underlying factors contributing to the bioreactor’s collapse and subsequent recovery, metagenomic analyses were performed on samples collected in December 2016, during the crash period, and February 2017, after the recovery had been initiated in January 2017. These two sampling dates enabled a comparison of reactor-associated microbial community composition and predicted functional potential between the crash and early recovery operational states.

The metagenomic sequencing of seven samples generated an initial dataset comprising approximately 98.6 million reads. Following the quality control process, a refined dataset was obtained, retaining 96.4 million (97.8%) sequences for further analysis.

Figure 2 illustrates the outcomes of the Principal Component Analysis (PCA) applied to the microbial community data. Figure 2A represents the PCA based on taxonomic profiles derived from KAIJU classifications, while Figure 2B presents the PCA based on functional profiles inferred from KEGG Orthology (KO) annotations using HUMAnN2. In both analyses, samples collected during the December 2016 system crash and those collected during the February 2017 recovery phase formed clearly separated clusters, indicating substantial differences in microbial community structure and functional potential between the two sampled operational states. Statistical testing confirmed these differences. Taxonomic profiles differed significantly between the two sampling periods (PERMANOVA, *R*^2^ = 0.86, *p* = 0.038; ANOSIM R = 1, *p* = 0.04), and functional profiles also showed significant separation (PERMANOVA, *R*^2^ = 0.61, *p* = 0.024; ANOSIM R = 0.93, *p* = 0.034).

**Figure 2:**
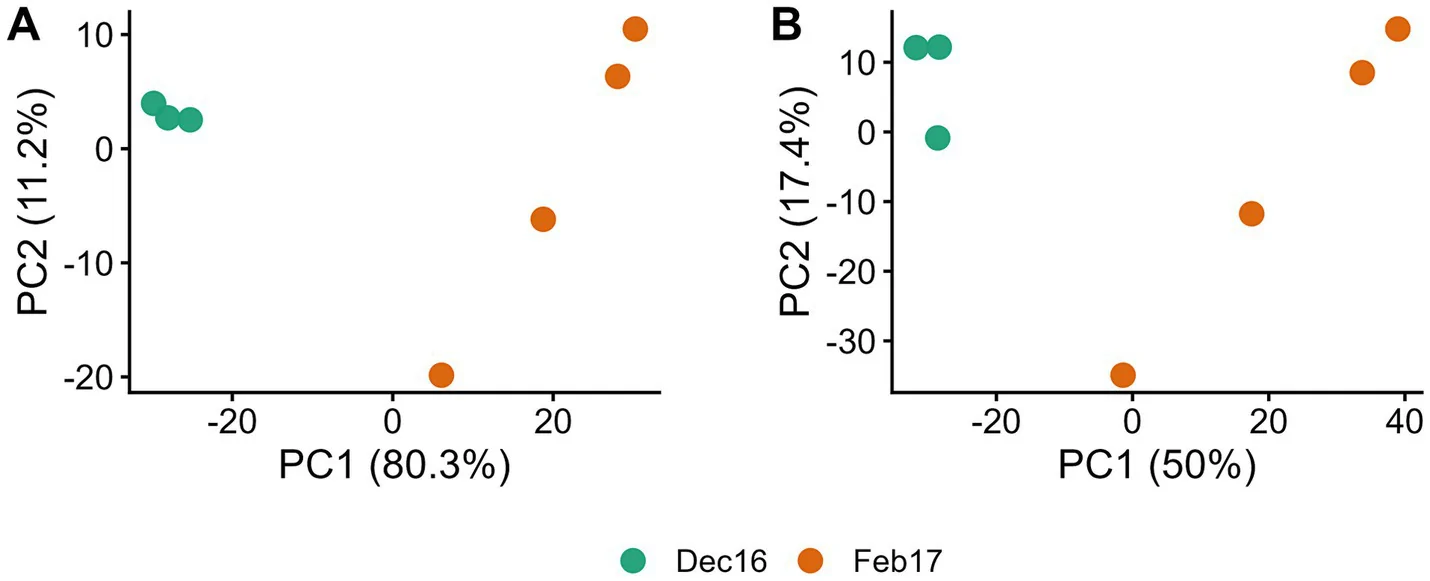
Principal component analysis (PCA) of the microbial community in terms of **(A)** taxonomy based on KAIJU and **(B)** functional pathways based on KO by HUMAnN2.

### 3.3 MAG reconstruction, quality, taxonomy, and abundance

The metagenomic analysis yielded a comprehensive set of 51 MAGs, each demonstrating a completeness exceeding 50% and contamination levels below 5%, as detailed in Supplementary Table S1. Figure 3 presents the abundance profiles of the significantly enriched Metagenome-Assembled Genomes (MAGs), expressed as genome copies per million reads and normalized for differences in MAG length. Only MAGs with an LDA score >3 and a Benjamini–Hochberg-adjusted *q*-value <0.05 are shown. The heatmap displays MAG abundance across the crash and recovery samples, while the accompanying bar plot shows the corresponding LEfSe LDA effect sizes and direction of enrichment. GTDB-based taxonomic assignments are indicated next to each MAG. Among the detected bins, bin4, bin24 and bin28 (class *Gammaproteobacteria*) and bin40 (class *Bacteroidia*) were the most abundant, while bin16, bin8, bin6 and bin5 (*Gammaproteobacteria*) and bin15 (*Bacteroidia*) also reached notable abundances. Furthermore, a distinct pattern in the distribution of these bins became evident. Specifically, bin4 and bin24 were more abundant in the December crash samples, whereas bin40 and bin28 were more abundant in the February early-recovery samples. This differentiation highlights contrasting MAG abundance patterns between the two sampled operational states.

**Figure 3:**
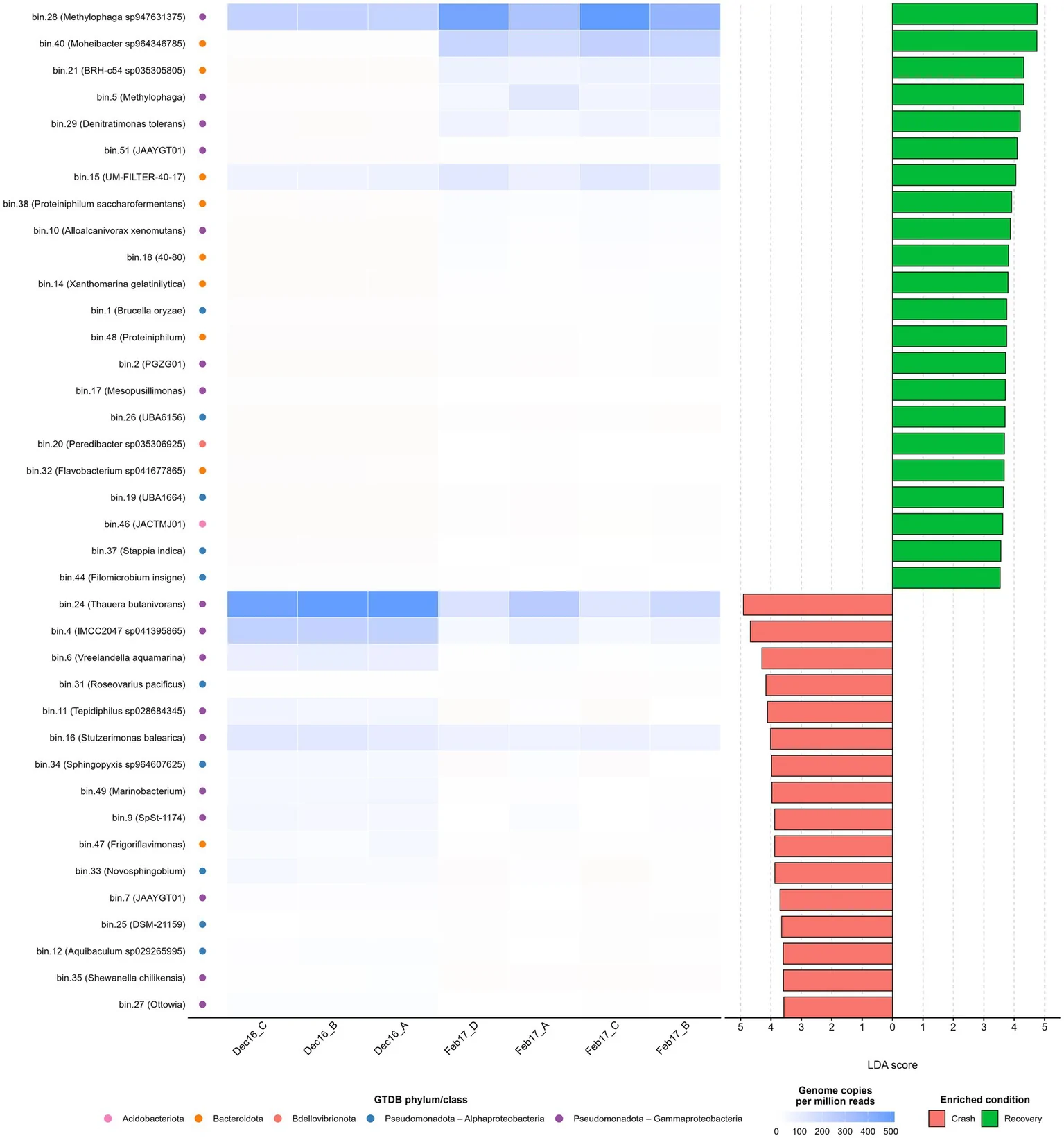
Differentially enriched Metagenome-Assembled Genomes (MAGs) identified by LEfSe. The heatmap shows the abundance of MAGs that passed the significance criteria of an LDA score >3 and a Benjamini–Hochberg-adjusted *q*-value <0.05, expressed as genome copies per million reads across the crash and early recovery samples. The color scale ranges from white to blue, with darker blue shades indicating higher abundance. Colored points next to the MAG labels indicate GTDB-based taxonomic affiliation, with Pseudomonadota separated by class. The bar plot shows the corresponding LEfSe LDA effect sizes and the direction of enrichment in the Crash or Recovery condition.

GTDB-Tk classified all 51 MAGs to at least family level, with no genome left unresolved above that rank: 25 MAGs (49%) were assigned at the species level, 24 (47%) at the genus level, and 2 (4%) at the family level (Supplementary Table S2). The 51 bacterial MAGs spanned five phyla, dominated by Pseudomonadota (39 MAGs; 24 *Gammaproteobacteria* and 15 *Alphaproteobacteria*) and *Bacteroidota* (9 MAGs), with single representatives of *Acidobacteriota, Bdellovibrionota* and *Desulfobacterota*. Notably, several genomes traditionally classified as *Betaproteobacteria* (e.g., *Thauera*, *Diaphorobacter* and *Achromobacter*) are placed by the GTDB within the *Gammaproteobacteria* as members of the order *Burkholderiales*, which partly accounts for the numerical dominance of this class. A phylogenomic tree of the 51 MAGs (Supplementary Figure S1) recovered genomes of the same GTDB class in coherent, well-supported clusters, with *Gammaproteobacteria*, *Alphaproteobacteria* and *Bacteroidota* each forming distinct groups. As the tree was inferred from a concatenated marker-protein alignment spanning multiple phyla, it was left unrooted; branch support was generally high within each class, whereas the deeper relationships between phyla were poorly resolved, and the rooting shown in Supplementary Figure S1 is therefore arbitrary and should not be interpreted evolutionarily. Among the enriched bins shown in Figure 3, most were resolved to genus or species level. bin24 was assigned to *Thauera butanivorans* (family *Rhodocyclaceae*, order *Burkholderiales*), bin28 to the genus *Methylophaga* (*Methylophaga* sp947631375, family Methylophagaceae), bin40 to the genus *Moheibacter* (*Moheibacter* sp964346785, family Weeksellaceae), and bin4 to the uncultured genus-level lineage IMCC2047 (IMCC2047 sp041395865, order *Pseudomonadales*).

### 3.4 Predicted functional traits of focal MAGs

Functional annotation revealed distinct predicted metabolic and genetic characteristics among the abundant bacterial bins associated with the two operational phases of the MBR. Bin4 and bin24, which increased in abundance during the crash period, contained genes and predicted pathways that may hypothetically be associated with persistence under deteriorating reactor conditions (Figures 4A,B). In contrast, bin28 and bin40 were more abundant during the recovery period and contained predicted functional traits that may hypothetically be associated with persistence under early recovery conditions (Figures 5A,B). These differences were identified based on the METABOLIC (Supplementary Table S6) and Prokka (Supplementary Table S5) annotation results.

**Figure 4:**
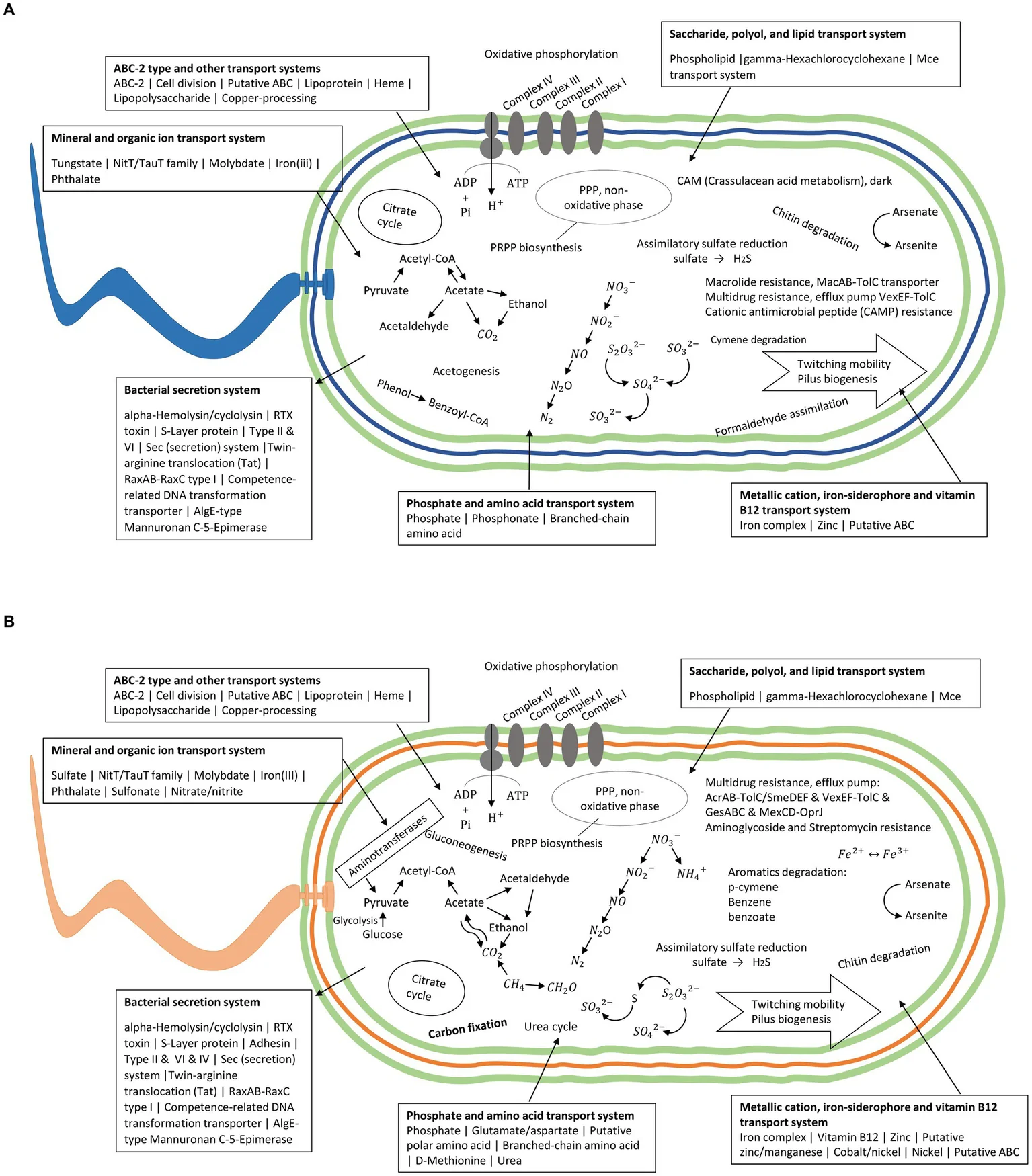
Schematic representations of bacterial cells: **(A)** for bin4 and **(B)** for bin24, which were enriched during the crash condition. Predicted functional traits were inferred from METABOLIC and Prokka annotations.

**Figure 5:**
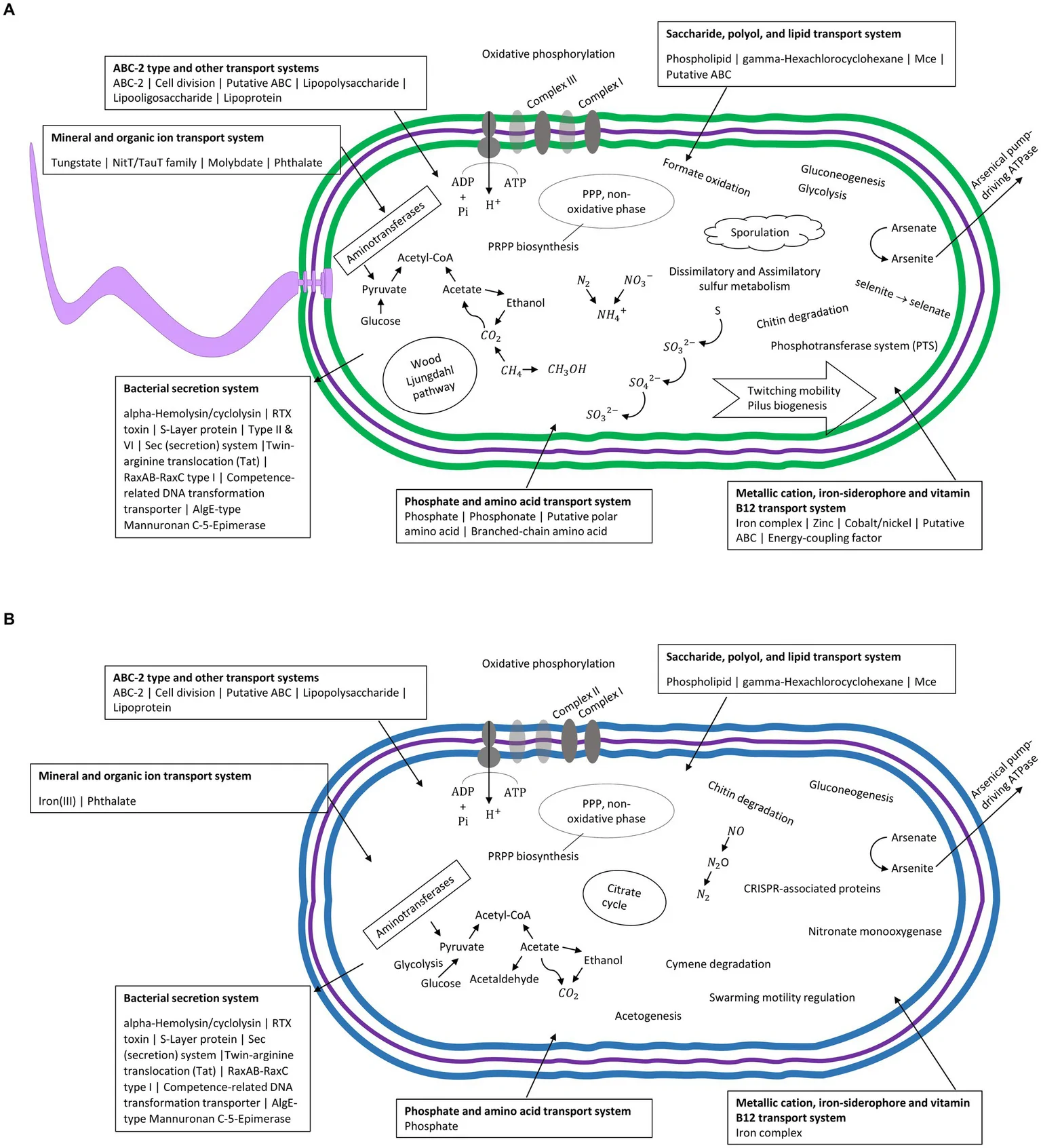
Schematic representations of bacterial cells: **(A)** bin28 and **(B)** bin40, which were enriched during the early recovery condition. Predicted functional traits were inferred from METABOLIC and Prokka annotations.

Collectively, the four focal MAGs contained genes annotated as being associated with arsenate reduction to arsenite, the non-oxidative phase of the pentose phosphate pathway (PPP), and phosphoribosyl pyrophosphate biosynthesis (PRPP). Bin4, bin24, and bin28 shared the presence of genes related to chitin degradation (hexosaminidase), assimilatory sulfate reduction and other sulfur cycle processes (*soxB, soxY, soxC, sdo, sat, sqr*). Among the focal MAGs, bin28 contained the broadest set of genes associated with sulfur oxidation, and dissimilatory and assimilatory sulfur metabolism.

While all four bins contained genes associated with nitrogen-cycle processes, distinct predicted functional profiles were observed among them. Notably, genes associated with nitrogen fixation were detected in bin28, whereas bin4 and bin24 contained genes associated with denitrification capabilities (*narG, nirS, nosZ, norB, norC*—all present in bin24, with some also in bin4). Bin24 additionally contained *napA* and *napB*, which are associated with nitrate reduction.

Bin4, bin24, and bin40 contained genes annotated as being associated with cymene degradation, indicating predicted genomic potential for the degradation of this aromatic compound. Additionally, bin24 showed a broader predicted potential for aromatic compound degradation, including cymene, benzene, and benzoate. Bin4 also encoded the predicted conversion of phenol to benzoyl-CoA.

Bin24 also contained genes associated with carbon fixation, urea cycle, iron oxidation and reduction, whereas bin4 contained genes associated with acetogenesis.

Bin4, bin24, and bin28 encoded putative markers associated with twitching motility and pilus biogenesis, whereas bin40 contained genes associated with the regulation of swarming motility and CRISPR-associated proteins.

Bin4 and bin24 contained genes representing the tricarboxylic acid cycle and all five oxidative-phosphorylation complexes, whereas bin28 and bin40 contained only subsets of the annotated genes associated with these systems. Bin4 and bin24 also contained genes associated with antimicrobial-resistance mechanisms, including multidrug efflux pumps. These annotations indicate predicted genomic potential and do not establish that the corresponding processes were active in the MBR at the time of sampling.

## 4 Discussion

The industrial wastewater treated by MBR systems contains many toxic compounds, high organic loads, and extreme physicochemical properties, making it necessary to apply an effective treatment for preventing environmental pollution. MBRs have emerged as robust solutions, offering efficient pollutant removal and high-quality effluent production due to the synergy between activated sludge processes and membrane filtration technologies (Rahman et al., 2023). The variability of microbial community composition within MBRs is influenced by the influent wastewater characteristics, operational conditions and reactor design, emphasizing the significance of understanding the microbial consortia’s roles in these systems (Al-Asheh et al., 2021; Nguyen et al., 2020). This study presents a genome-resolved comparison of microbial community composition and predicted functional potential between two sampled operational states of an industrial wastewater treatment MBR system: crash and early recovery. The analysis identifies differences in MAG abundance and in genes associated with metabolic and biogeochemical processes between the two sampled states.

The prolonged deterioration in reactor performance and subsequent early recovery phase highlighted the MBR system’s vulnerability to operational disruptions. This crash condition was manifested as a substantial decline in TOC removal efficiency coupled with a pronounced increase in effluent BOD (Figure 1). The increase in effluent BOD could be attributed to inhibition of the biodegradation process, causing an accumulation of degraded biomass which resulted in accessible organic matter in the effluent (Yogalakshmi and Joseph, 2010). During the subsequent recovery phase, a gradual improvement in treatment efficacy was observed, suggesting the system’s resilience and potential for restoration. These findings emphasize the necessity for predictive maintenance and early warning systems in MBR operations, enabling proactive measures to mitigate crashes and ensure consistent performance (Niya et al., 2024). To allow a deeper understanding of the microbial community dynamics underlying these operational shifts, a metagenomic analysis was performed.

Metagenomic approaches have significantly transformed our understanding of microbial ecosystems, enabling detailed insights into microbial diversity and functionality in various environments, including industrial wastewater treatment systems (Niya et al., 2024). The advancements in sequencing technologies and bioinformatics tools have enhanced the sensitivity and accuracy of metagenomic analyses, addressing previous challenges such as data completeness and the lack of reference genomes for diverse microorganisms. This study builds upon these innovations, showcasing the value of metagenomics in unraveling the roles of microbial communities under stress conditions and during recovery phases in MBR systems.

The principal component analyses (PCA) (Figure 2) of taxonomic and functional profiles revealed distinct separation between microbial communities during the crash and recovery periods. Likewise, earlier research has also established a connection between microbial community composition and operational parameters of wastewater treatment systems, including factors such as temperature, BOD, TOC, PO_4_, NH_4_+-N, and TN (Barak et al., 2020; Lee et al., 2016; Liu et al., 2016).

To elucidate the metabolic and genetic potential of key microbial populations associated with the observed community differences, Metagenome-Assembled Genomes (MAGs) were reconstructed and annotated. Among the prominent MAGs, notable differences in abundance were observed for specific bins: bin4 and bin24 during the crash period, as well as bin28 and bin40 during the early recovery period (Figure 3). These MAGs exhibited distinct enrichment patterns between the two operational states, suggesting possible associations with the contrasting reactor conditions. Importantly, these MAGs contained genes associated with diverse metabolic processes potentially relevant to industrial wastewater treatment (Figures 4, 5).

The genome-resolved taxonomy places the MBR community within lineages that typically dominate activated-sludge and MBR systems, where Pseudomonadota and Bacteroidota are consistently the most abundant phyla (Wu et al., 2019). Beyond this broad pattern, the GTDB classification resolved several genomes to genera whose known physiologies are consistent with the functional potential described above. The prevalence of several halophilic or marine-associated genera, including *Methylophaga*, *Alloalcanivorax*, *Vreelandella*, and *Marinobacterium*, is consistent with the high salinity of the treated industrial wastewater, for which the mean raw TDS concentration was approximately 22,000 mg/L. Such conditions likely favored salt-adapted microbial populations and may have contributed to shaping the observed community composition (Tan et al., 2019). The crash-enriched bin24 was assigned to *Thauera*, a genus of denitrifiers well known for degrading a wide range of aromatic compounds—including phenol, toluene and benzoate derivatives—under both oxic and denitrifying conditions, and regarded as functionally important in industrial wastewater treatment (Mao et al., 2010; Mechichi et al., 2002); this is consistent with the denitrification- and aromatic-degradation-associated genes detected in bin24 and may also be compatible with lower-oxygen conditions during the high-load crash period, although oxygen availability was not directly measured. Among the alphaproteobacterial genomes, the Sphingomonadales (*Novosphingobium*, bin33/bin45; *Sphingopyxis*, bin34) are recognized degraders of recalcitrant mono- and polycyclic aromatic and xenobiotic compounds (Kertesz and Kawasaki, 2010; Stolz, 2009), while the *Bacteroidota* genomes included *Proteiniphilum saccharofermentans* (bin38), a proteolytic, fermentative member of the phylum (Hahnke et al., 2016). The recovery-enriched bin28 was classified as *Methylophaga*, a genus of moderately halophilic marine methylotrophs that oxidise one-carbon compounds such as methanol and methylamine (Neufeld et al., 2007), consistent with the C1-related genes detected in this genome and with its enrichment during a recovery phase that may have been relatively more oxic; however, oxygen availability was not directly measured and this interpretation remains speculative.

These genome-resolved findings can also be related to the community-level diversity patterns previously reported for this same MBR system (Barak et al., 2020), where bacterial richness (Chao1, Observed OTUs) and diversity (Shannon, PD Whole Tree) were both significantly lower during the December 2016 crash than in the following recovery months. Because the present MAGs were co-assembled across all samples rather than estimated per sample, no equivalent MAG-level diversity index was calculated here, so this decline in overall richness cannot be directly re-tested at the genome-resolved level. Nevertheless, several taxa distinguishing the crash and recovery conditions in the present analysis corroborate the earlier compositional shifts: the crash-enriched bin24 (*Thauera butanivorans*) and the recovery-enriched bin28 (*Methylophaga*) and bin40 (*Moheibacter*) correspond to the same genera previously reported as enriched in the respective periods. The genome-resolved data thus extend these earlier community-level patterns by linking the corroborating taxa to their predicted functional gene content, as detailed above.

Several functional attributes were observed across the MAGs, with genomic annotations suggesting potential involvement in critical biogeochemical cycles related to sulfur, nitrogen and carbon cycling (Grubba et al., 2022). These cycles are involved in pollutant transformation and removal, including the conversion of harmful compounds into less toxic forms. Indeed, bin4 and bin24 contained genes associated with denitrification capabilities, consistent with their increased occurrence during the crash period when the MBR conditions were assumed to be more anoxic; however, oxygen availability was not directly measured. In line with this hypothesis, the subsequent recovery period may have involved relatively more oxic conditions; however, this interpretation remains speculative, and the MAGs contained genes associated with multiple nitrogen-cycle processes, including denitrification, nitrification, and nitrogen fixation (Fu et al., 2009). The inclusion of genes associated with sulfur oxidation, assimilatory sulfate reduction, and similar processes within the MAGs suggests genomic potential for sulfur metabolism.

Moreover, the predicted genomic potential of these microbial populations to degrade recalcitrant aromatic compounds such as cymene, benzene, phenol, and benzoate is of particular interest. The presence of genes associated with the degradation of these complex compounds suggests potential relevance to industrial wastewater containing diverse aromatic pollutants (Mozo et al., 2011). Recent metagenomic analyses of industrial activated sludge have similarly resolved the distribution and hosts of degradation genes for a wide range of aromatic pollutants, demonstrating that xenobiotic metabolism is often partitioned across multiple bacterial lineages within the community (Gao et al., 2024). Our genome-resolved results extend these observations to a full-scale industrial MBR by linking predicted aromatic-degradation traits, such as cymene, benzene, and phenol catabolism, to individual MAGs that showed distinct enrichment patterns during crash and early recovery conditions.

Additionally, the presence of resistance-related genes suggests predicted genomic potential for tolerance to inhibitory compounds that may be present in the wastewater.

The functional analysis conducted in this study is rooted in DNA-based assessments, which provide insights into the potential capabilities of the microbial community. For functional analysis, we utilized multiple pipelines based on different databases. Therefore, discrepancies between the results may arise, and caution must be taken when analyzing and interpreting the data. It is also important to note that this approach indicates the genetic potential for specific activities rather than the actual activity profile. To delve into the active functional roles, metatranscriptomics becomes indispensable (Mukherjee and Reddy, 2020). Metatranscriptomic analyses enable the exploration of gene expression patterns, offering a more comprehensive understanding of the active functions within the microbial community in each operational state.

The application of GTDB-Tk substantially improved the taxonomic resolution of the recovered MAGs, allowing all genomes to be classified to at least the family level and most to the genus or species level. Nevertheless, taxonomic assignments alone provide only limited insight into the ecological roles of these populations. By combining genome-resolved taxonomy with functional annotation, the present metagenomic approach enabled the association of individual microbial populations with predicted metabolic traits potentially relevant to industrial wastewater treatment. This is particularly valuable for microbial populations that remain poorly characterized or difficult to cultivate, and highlights the contribution of genome-resolved metagenomics to the study of complex MBR communities.

## 5 Conclusion

In summary, this study identified differences in microbial community composition and predicted genomic potential between the crash and early recovery conditions of an industrial MBR system. The metagenomic analysis provided a genome-resolved view of the predicted functional potential of the microbial populations detected in the system. Specifically, genes associated with nitrogen cycling, sulfur metabolism, and organic matter degradation were identified in several MAGs and may represent functional potential relevant to industrial wastewater treatment. These findings provide hypotheses that may inform future studies of microbial indicators associated with MBR operational status, stability, and recovery.

Future research efforts could delve deeper into the functional interrelationships between microbial populations, explore the intricate molecular mechanisms underlying the observed capabilities, and investigate strategies for harnessing and enhancing the beneficial traits of key microbial players to further improve MBR system resilience and efficiency.

### 5.1 Limitations of the study

One notable limitation of this study relates to the absence of sampling conducted prior to the bioreactor crash and after its recovery. Due to logistical constraints, the opportunity to gather data before the incident was regrettably missed. In addition, the study was based on only two sampling dates and a relatively small number of samples, with operational state fully confounded with sampling date. The samples representing each operational condition were collected from sequential reactors within the treatment system and therefore do not constitute fully independent biological replicates. These limitations may have implications for fully understanding the temporal dynamics and complete impact of the crash on the bioreactor microbial community. In addition, direct measurements confirming oxic or anoxic conditions were not available for the sampled periods; therefore, interpretations regarding oxygen conditions should be considered speculative rather than directly demonstrated.

## Data Availability

The datasets associated with this study have been deposited in the National Center for Biotechnology Information (NCBI) database under BioProject ID PRJNA1033225.

## Supplementary material

SUPPLEMENTARY FIGURE S1 Maximum-likelihood phylogenomic tree of the consolidated metagenome-assembled genomes (MAGs), constructed from the GTDB-Tk bac120 concatenated marker-protein alignment using IQ-TREE version 3.1.2. Branch support values represent SH-aLRT and ultrafast bootstrap support based on 1,000 replicates each. Taxonomic assignments were determined using GTDB-Tk version 2.7.2 with GTDB release R232. The tree was visualized and annotated in R using ggtree version 4.2.0.
